# Supplementary material for: Evaluating the modulation of peripheral immune profile in people living with HIV and (Neuro)cysticercosis
Source: PLoS Negl Trop Dis. 2024 Aug 2;18(8):e0012345. doi: 10.1371/journal.pntd.0012345 (PMC11324146; doi:10.1371/journal.pntd.0012345)
Supplement: S1 Text — (DOCX) [file pntd.0012345.s001.docx]

**Table 1. Baseline demographic and clinical characteristics of study participants, stratified by HIV status.**

| **Variables** | **Total**  **n (%)** | **HIV Positive**  **n (%)** | **HIV Negative**  **n (%)** | ***p-value**** |
| --- | --- | --- | --- | --- |
|  | 234 (100) | 110 | 124 |  |
| **Region** |  |  |  |  |
| Iringa | 67 (28.6) | 33 (30.0) | 34 (27.4) | 0.695 |
| Mbeya | 167 (71.4) | 77 (70.0) | 90 (72.6) |  |
| Median age years (IQR) |  | 43(17) | 42(16) |  |
| **Age group. (years)** |  |  |  |  |
| 13 - 24 | 4(1.7) | 0(0.0) | 4(3.2) | 0.189 |
| 25 - 34 | 49(20.9) | 19(17.3) | 30(24.2) |  |
| 35 – 44 | 73(31.2) | 36(32.7) | 37(29.8) |  |
| 45 - 54 | 61(26.1) | 30(27.3) | 31(25.0) |  |
| 55+ | 47(20.1) | 25(22.7) | 22(17.7) |  |
| **Sex** |  |  |  |  |
| Male | 152 (65.0) | 70 (63.6) | 82 (66.1) | 0.728 |
| Female | 82 (35.0) | 40 (36.4) | 42 (33.9) |  |
| **Marital status** |  |  |  |  |
| Married | 171 (73.1) | 79 (71.8) | 92 (74.2) | 0.002 |
| Single | 21 (9.0) | 4 (3.6) | 17 (13.7) |  |
| Unmarried**^⁑^** | 42 (17.9) | 27 (24.6) | 15 (12.1) |  |
| **Education Level** |  |  |  |  |
| Informal | 24 (10.3) | 13 (11.8) | 11 (8.9) | 0.789 |
| Primary | 54 (23.1) | 26 (23.6) | 28 (22.6) |  |
| Secondary | 144 (61.5) | 66 (60.0) | 78 (62.9) |  |
| Post-secondary | 12 (5.1) | 5 (4.6) | 7 (5.6) |  |
| **Occupation** |  |  |  |  |
| Formal Employment | 22(9.4) | 6(5.5) | 16(12.9) | 0.256 |
| Unemployed | 19(8.1) | 7(6.4) | 12(9.7) |  |
| Miners | 10(4.3) | 5(4.5) | 5(4.0) |  |
| Small Business | 9(3.8) | 4(3.6) | 5(4.0) |  |
| Farmers | 174(74.4) | 88(80.0) | 86(69.4) |  |
| **Markers of HIV Progression** | | | | |
| **CD4 counts (cells/ µL)** |  |  |  |  |
| <200 | 33 (30.0) | 33 (30.0) | N/A | N/A |
| 200 -500 | 38 (34.5) | 38 (34.5) | N/A |  |
| >500 | 39 (34.5) | 39 (34.5) | N/A |  |
| **Months on HAART** |  |  |  |  |
| < 6 | 30 (27.2) | 30 (27.2) | N/A | N/A |
| 6 – 12 | 6 (5.5) | 6 (5.5) | N/A |  |
| 12 - 24 | 6 (5.5) | 6 (5.5) | N/A |  |
| > 24 | 68 (61.8) | 68 (61.8) | N/A |  |
| **HIV viral load (copies/ml)** |  |  |  |  |
| Undetectable | 80(72.7) | 80(72.7) | N/A | N/A |
| <1000 | 25(22.7) | 25(22.7) | N/A |  |
| >1000 | 5(4.6) | 5(4.6) | N/A |  |
| **HIV/AIDS Clinical Stage** |  |  |  |  |
| Stage I | 43(39.1) | 43(39.1) | N/A | N/A |
| Stage II | 22(20.0) | 22(20.0) | N/A |  |
| Stage III | 37(33.6) | 37(33.6) | N/A |  |
| Stage IV | 7(6.4) | 7(6.4) | N/A |  |
| Missing | 1(0.9) | 1(0.9) | N/A |  |

***P-values were calculated using the chi-square test; p<0.05 is considered significant. ⁑ Includes (divorced and widowed).**
