# Supplementary material for: Evaluating the modulation of peripheral immune profile in people living with HIV and (Neuro)cysticercosis
Source: PLoS Negl Trop Dis. 2024 Aug 2;18(8):e0012345. doi: 10.1371/journal.pntd.0012345 (PMC11324146; doi:10.1371/journal.pntd.0012345)
Supplement: S2 Text — (DOCX) [file pntd.0012345.s002.docx]

**Table 2. Multiple regression analysis of age, sex, HIV status, and NCC status on cytokine levels***

| **Cytokine** | **Variables** | **β (SE)** | **95% CI** | **p-value** |
| --- | --- | --- | --- | --- |
| **TNF-α _log** | Age (years) | -0.002 (0.006) | (-0.014,0.01) | 0.748 |
|  | Sex (Male =1) | -0.179 (0.14) | (-0.454,0.097) | 0.203 |
|  | CC (Positive=1) | 0.171 (0.193) | (-0.209,0.551) | 0.377 |
|  | HIV (Positive=1) | 0.614 (0.179) | (0.262,0.966) | 0.001 |
|  | CC x HIV | -0.574 (0.271) | (-1.108,-0.04) | 0.035 |
|  |  |  |  |  |
| **IL-8_log** | Age (years) | 0.01 (0.013) | (-0.015,0.035) | 0.448 |
|  | Sex (Male =1) | -0.737 (0.303) | (-1.334,-0.14) | 0.016 |
|  | CC (Positive=1) | 0.065 (0.417) | (-0.758,0.887) | 0.877 |
|  | HIV (Positive=1) | 1.074 (0.387) | (0.312,1.836) | 0.006 |
|  | CC x HIV | -0.9 (0.586) | (-2.055,0.256) | 0.126 |
|  |  |  |  |  |
| **IL-1β _log** | Age (years) | 0.002 (0.006) | (-0.009,0.014) | 0.71 |
|  | Sex (Male =1) | -0.175 (0.141) | (-0.453,0.103) | 0.216 |
|  | CC (Positive=1) | -0.152 (0.194) | (-0.535,0.231) | 0.435 |
|  | HIV (Positive=1) | 0.385 (0.18) | (0.031,0.74) | 0.033 |
|  | CC x HIV | -0.311 (0.273) | (-0.849,0.226) | 0.255 |
|  |  |  |  |  |
| **IFN-γ _log** | Age (years) | -0.005 (0.004) | (-0.013,0.004) | 0.263 |
|  | Sex (Male =1) | -0.127 (0.102) | (-0.329,0.075) | 0.216 |
|  | CC (Positive=1) | 0.254 (0.141) | (-0.024,0.532) | 0.073 |
|  | HIV (Positive=1) | 0.441 (0.131) | (0.183,0.698) | 0.001 |
|  | CC x HIV | -0.577 (0.198) | (-0.967,-0.187) | 0.004 |
|  |  |  |  |  |
| **IL-17_log** | Age (years) | 0.004 (0.005) | (-0.005,0.013) | 0.402 |
|  | Sex (Male =1) | -0.279 (0.11) | (-0.495,-0.063) | 0.012 |
|  | CC (Positive=1) | -0.079 (0.151) | (-0.376,0.219) | 0.603 |
|  | HIV (Positive=1) | 0.301 (0.14) | (0.026,0.577) | 0.032 |
|  | CC x HIV | -0.044 (0.212) | (-0.462,0.374) | 0.836 |
|  |  |  |  |  |
| **IL-12_log** | Age (years) | -0.001 (0.006) | (-0.013,0.012) | 0.92 |
|  | Sex (Male =1) | -0.066 (0.152) | (-0.366,0.234) | 0.665 |
|  | CC (Positive=1) | 0.271 (0.21) | (-0.143,0.685) | 0.198 |
|  | HIV (Positive=1) | 0.539 (0.194) | (0.155,0.922) | 0.006 |
|  | CC x HIV | -0.533 (0.295) | (-1.114,0.048) | 0.072 |
|  |  |  |  |  |
| **IL-18_log** | Age (years) | -0.005 (0.003) | (-0.011,0.002) | 0.189 |
|  | Sex (Male =1) | -0.081 (0.083) | (-0.244,0.082) | 0.33 |
|  | CC (Positive=1) | 0.025 (0.114) | (-0.2,0.25) | 0.828 |
|  | HIV (Positive=1) | -0.262 (0.106) | (-0.47,-0.053) | 0.014 |
|  | CC x HIV | -0.049 (0.16) | (-0.365,0.267) | 0.76 |
|  |  |  |  |  |
| **IL-6_log** | Age (years) | 0.014 (0.009) | (-0.003,0.031) | 0.113 |
|  | Sex (Male =1) | -0.383 (0.204) | (-0.785,0.019) | 0.062 |
|  | CC (Positive=1) | -0.139 (0.281) | (-0.692,0.415) | 0.622 |
|  | HIV (Positive=1) | 0.688 (0.26) | (0.175,1.201) | 0.009 |
|  | CC x HIV | -0.485 (0.395) | (-1.263,0.292) | 0.22 |
|  |  |  |  |  |
| **IL-10_log** | Age (years) | 0.005 (0.006) | (-0.006,0.016) | 0.391 |
|  | Sex (Male =1) | -0.059 (0.132) | (-0.318,0.201) | 0.657 |
|  | CC (Positive=1) | -0.419 (0.181) | (-0.776,-0.062) | 0.022 |
|  | HIV (Positive=1) | 0.012 (0.168) | (-0.319,0.343) | 0.943 |
|  | CC x HIV | 0.12 (0.255) | (-0.381,0.622) | 0.637 |
|  |  |  |  |  |
| **IL-4_log** | Age (years) | 0.002 (0.007) | (-0.013,0.016) | 0.818 |
|  | Sex (Male =1) | -0.483 (0.176) | (-0.831,-0.136) | 0.007 |
|  | CC (Positive=1) | -0.382 (0.243) | (-0.861,0.097) | 0.118 |
|  | HIV (Positive=1) | 0.439 (0.225) | (-0.005,0.883) | 0.053 |
|  | CC x HIV | 0.283 (0.341) | (-0.39,0.956) | 0.408 |
|  |  |  |  |  |
| **IL-5_log** | Age (years) | -0.002 (0.003) | (-0.009,0.004) | 0.532 |
|  | Sex (Male =1) | 0.034 (0.079) | (-0.121,0.188) | 0.669 |
|  | CC (Positive=1) | 0.229 (0.108) | (0.016,0.442) | 0.035 |
|  | HIV (Positive=1) | 0.254 (0.1) | (0.056,0.451) | 0.012 |
|  | CC x HIV | -0.5 (0.152) | (-0.799,-0.2) | 0.001 |
|  |  |  |  |  |
| **IL-13_log** | Age (years) | 0.004 (0.003) | (-0.001,0.01) | 0.129 |
|  | Sex (Male =1) | -0.007 (0.066) | (-0.137,0.124) | 0.918 |
|  | CC (Positive=1) | -0.107 (0.091) | (-0.287,0.073) | 0.243 |
|  | HIV (Positive=1) | 0.041 (0.085) | (-0.126,0.208) | 0.628 |
|  | CC x HIV | 0.068 (0.128) | (-0.185,0.32) | 0.599 |
|  |  |  |  |  |
| **VCAM-1_log** | Age (years) | -0.005 (0.005) | (-0.015,0.005) | 0.289 |
|  | Sex (Male =1) | 0.042 (0.118) | (-0.191,0.274) | 0.724 |
|  | CC (Positive=1) | 0.262 (0.163) | (-0.059,0.583) | 0.109 |
|  | HIV (Positive=1) | 0.047 (0.151) | (-0.25,0.344) | 0.754 |
|  | CC x HIV | -0.603 (0.229) | (-1.053,-0.152) | 0.009 |
|  |  |  |  |  |
| **ICAM-1_log** | Age (years) | -0.008 (0.009) | (-0.026,0.01) | 0.38 |
|  | Sex (Male =1) | -0.535 (0.213) | (-0.954,-0.116) | 0.013 |
|  | CC (Positive=1) | 0.405 (0.293) | (-0.172,0.982) | 0.168 |
|  | HIV (Positive=1) | 0.126 (0.271) | (-0.409,0.66) | 0.644 |
|  | CC x HIV | -0.032 (0.411) | (-0.843,0.779) | 0.938 |

***The P-value* assesses the statistical significance of the regression coefficient. * The “_log” after each cytokine reflects the log-transformation of variables to make them normally distributed**
