# Supplementary material for: Evaluating the modulation of peripheral immune profile in people living with HIV and (Neuro)cysticercosis
Source: PLoS Negl Trop Dis. 2024 Aug 2;18(8):e0012345. doi: 10.1371/journal.pntd.0012345 (PMC11324146; doi:10.1371/journal.pntd.0012345)
Supplement: Attachment — Submitted filename: Comments- HIV+ NCC coinfection paper.docx [file pntd.0012345.s005.docx]

**Evaluating the modulation of peripheral immune profile in people living with HIV and Neurocysticercosis**

The present study is interesting and data about HIV and NCC co-infection is very less available, hence this study will definitely will be of interest to community. However, manuscript in its present format is difficult to understand and need major revision.

**Major Comments-**

- They should discuss the sensitivity of Antigen/ Antibody ELISA and the western blot used in the study, as right diagnosis of NCC is a major concern. In the present study too cysticercosis Ag positivity was 35.5% but on CT only 20% were positive.
- They used CT (not MRI) for radio-diagnosis of NCC, they should discuss the limitation of this approach.

Prasad KN, Prasad A, Gupta RK, Nath K, Pradhan S, Tripathi M, Pandey CM. Neurocysticercosis in patients with active epilepsy from the pig farming community of Lucknow district, north India. Trans R Soc Trop Med Hyg. 2009 Feb;103(2):144-50. doi: 10.1016/j.trstmh.2008.07.015.

- The authors should discuss the presence of asymptomatic NCC cases in the community and its implication in the context of the present study

Prasad A, Gupta RK, Pradhan S, Tripathi M, Pandey CM, Prasad KN. What triggers seizures in neurocysticercosis? A MRI-based study in pig farming community from a district of North India. Parasitol Int. 2008 Jun;57(2):166-71. doi: 10.1016/j.parint.2007.12.001.

- The authors should ensure that the Fig-1, 5 are cited properly within the context. (for example- In fig-1, you are showing fig J as a pro-inflammatory cytokine but actually fig J corresponds to IL-4, an anti-inflammatory cytokine).
- Explain and share the data in tabular form with mean and SD- how the authors are claiming significantly lower median concentration of IL-5 (Fig-2K), however it is not reflecting by the data attached in the manuscript. In fact this is observed for several cytokines.
- What could be the possible therapeutic targets in case of HIV and NCC co-infection. This aspect should be included in the manuscript.

The authors should include the challenges associated with diagnosis, treatment and control of the disease and how their finding can help in achieving this objective

Hossain MS, Shabir S, Toye P, Thomas LF, Falcone FH. Insights into the diagnosis,

vaccines, and control of Taenia solium, a zoonotic, neglected parasite. Parasit Vectors. 2023 Oct 24;16(1):380. doi: 10.1186/s13071-023-05989-6.

**Minor Comments-**

- Follow the scientific nomenclature to write the scientific names- See the line number 34 in the abstract.
- Check the citation style**-** one style should be used throughout the manuscript
- Re-check the data for any typological error
