# Supplementary material for: Evaluating the modulation of peripheral immune profile in people living with HIV and (Neuro)cysticercosis
Source: PLoS Negl Trop Dis. 2024 Aug 2;18(8):e0012345. doi: 10.1371/journal.pntd.0012345 (PMC11324146; doi:10.1371/journal.pntd.0012345)
Supplement: Attachment — Submitted filename: Response to Reviewers.docx [file pntd.0012345.s006.docx]

**Point-by-point responses to reviewers’ comments**

**Manuscript title:** Evaluating the modulation of peripheral immune profile in People Living with HIV and Neurocysticercosis.

**Manuscript Number:** PNTD-D-23-01498

The present study is interesting and data about HIV and NCC co-infection is very less available, hence this study will be of interest to community. However, manuscript in its present format is difficult to understand and need major revision.

**Reviewer #1 Comments and Responses**

**Major Comments:**

**Comment 1:** They should discuss the sensitivity of Antigen/ Antibody ELISA and the western blot used in the study, as right diagnosis of NCC is a major concern. In the present study too cysticercosis Ag positivity was 35.5% but on CT only 20% were positive.

Prasad KN, Prasad A, Gupta RK, Nath K, Pradhan S, Tripathi M, Pandey CM. Neurocysticercosis in patients with active epilepsy from the pig farming community of Lucknow district, north India. Trans R Soc Trop Med Hyg. 2009 Feb;103(2):144-50. doi: 10.1016/j.trstmh.2008.07.015.

**Response:** Thank you for highlighting the importance of discussing the diagnostic tools used in our study. We have now expanded the discussion on the sensitivity and specificity of the antigen and antibody ELISAs and Western blot assays utilized. This includes a detailed examination of their roles in diagnosing NCC, their limitations, and the implications of these limitations for our study findings. This information has been added to the Introduction Section, page 3 (lines 140-146), which now reads:

"Diagnosing Neurocysticercosis (NCC) accurately remains challenging due to its often-asymptomatic nature or presentation with a spectrum of non-specific, sometimes severe neurological symptoms. Diagnostic variability is evident in antibody and antigen ELISA sensitivities across different sample matrices, disease stages, and cyst characteristics, with sensitivities for the Ab-ELISA reported between less than 50% and up to 75%. While Ab-ELISA is valued for its high specificity in detecting T. solium antibodies and confirming exposure, it fails to differentiate between active, inactive, and past infections, potentially remaining positive after the parasite's viability ceases."

Introduction Section, pg.3 (lines 140 - 146), Discussion, pg. 17 (677 – 692) and Limitations section, pg. 20 (line 759-766) sections.

**Comment 2:** They used CT (not MRI) for radio-diagnosis of NCC, they should discuss the limitation of this approach.

**Response:** We appreciate your point on the limitations of using CT scans for diagnosing NCC. We have elaborated on why CT was chosen over MRI in our setting and discussed the limitations associated with this choice, particularly in detecting active cysts or cysts in certain brain regions. These details have been added to the Discussion, page 17 (lines 677-692) and Limitations section, page 20 (lines 759-766). The revised text in the Discussion section is:

"The cysticercosis Ag-ELISA detects circulating antigens indicative of viable cysts or active cysticercosis infection. While CT scans are highly specific for detecting the presence of cysts, they are not as effective as MRI in detecting active cysts or cysts in certain regions of the brain. Higher antigen positivity rates compared to CT scan confirmation could be due to several reasons including subclinical infections or very early stages of the disease that are not yet visible on CT scans. This likely explains our observation of antigen positivity in cases with no indicative CT scan findings."

**And in the Limitations section:**

"We acknowledge limitations in our imaging approach. The preference for CT scans (due to their wider availability in our region) over MRI may lead to underestimation of active cyst prevalence and missing cysts outside the brain."

These additions should comprehensively address the concerns you raised and have been highlighted as track changes in the manuscript for easy reference.

**Comment 3:** The authors should discuss the presence of asymptomatic NCC cases in the community and its implication in the context of the present study.

Prasad A, Gupta RK, Pradhan S, Tripathi M, Pandey CM, Prasad KN. What triggers seizures in neurocysticercosis? A MRI-based study in pig farming community from a district of North India. Parasitol Int. 2008 Jun;57(2):166-71. doi: 10.1016/j.parint.2007.12.001.

**Response:** Thank you very much for this insightful comment. We have expanded our discussion regarding the prevalence and implications of asymptomatic NCC within the community as it relates to our study findings.

Placement in the Manuscript: Discussion Section, page 17, Line 6770 to 692.

"In this study, we have considered the significant public health challenge posed by asymptomatic NCC cases. Asymptomatic infections often go undiagnosed due to the non-specific nature of their clinical presentation, which can complicate efforts to accurately gauge community prevalence and disease burden. Our findings, aligning with those of Schmidt et al. and Serpa et al., indicated no significant differences in the presence of cysticercosis antibodies or in the serological, clinical, and radiological presentations among HIV+ and HIV- patients co-infected with NCC. However, a notable discrepancy was observed between antigen positivity and confirmed CT scan findings, highlighting the limitations of current diagnostic methods. These methods show variable sensitivity and specificity, particularly when identifying cysts in certain brain regions or at different stages of NCC, such as active cases in the subarachnoid space or intraventricular areas. Given these diagnostic challenges, MRI is suggested as a superior alternative for detecting cysts in complex areas like the basal ganglia and brainstem, where CT scans are less effective. This study's findings underscore the importance of integrating comprehensive screening strategies, including serological testing, advanced imaging, and epidemiological assessments, to more effectively identify and manage both symptomatic and asymptomatic NCC cases."

**Further Clarifications:**

This expanded discussion aims to emphasize the complexity and implications of detecting and managing asymptomatic NCC within communities, particularly in resource-limited settings where advanced imaging techniques are not readily available. By integrating these points into the discussion, the manuscript addresses the critical need for improved diagnostic and management strategies that can capture the full spectrum of NCC, enhancing disease control and prevention efforts.

**Comment 4**: The authors should ensure that the Fig-1, 5 are cited properly within the context. (For example- In fig-1, you are showing fig J as a pro-inflammatory cytokine but actually fig J corresponds to IL-4, an anti-inflammatory cytokine).

Explain and share the data in tabular form with mean and SD- how the authors are claiming significantly lower median concentration of IL-5 (Fig-2K), however it is not reflecting by the data attached in the manuscript. In fact, this is observed for several cytokines.

**Response:** Thank you very much for spotting this. The Letter J was indeed a typo mistake. and has now been removed. See new Figure 1, page. 11.

Also, we do agree that we should only claim significance in data that reflect such statistically and in their central tendencies. Initially, we claimed a lower significance in IL-5 levels in CC+ than CC- individuals since the range values (IQR) were significantly lower for CC+ than CC- people (p=0.035), despite both groups having similar median values. We have now only described cases where the changes were apparent and statistically significant. For clarity, median values have been indicated in the text as much as possible. Also as suggested, we have shared the data for all figures in tabular forms with median and IQR for your review (attached with this response). We have included median and IQR instead of mean and SD since our data is not normally distributed.

Tables are included in the end of this point by point response to reviewers.

**Comment 5:** What could be the possible therapeutic targets in case of HIV and NCC co-infection. This aspect should be included in the manuscript.

**Response:** Thank you for this valuable suggestion. We have expanded our discussion to include a detailed examination of potential therapeutic targets that could address the complex interplay between HIV and NCC, particularly focusing on the observed immune response dynamics in co-infected patients.

**Placement in Manuscript:** Discussion Section, After Line 748 to 757

" This study addresses a critical gap in the literature concerning the peripheral immune responses to HIV and NCC coinfection. The findings highlight the need for a targeted approach in managing such coinfections, emphasizing the role of tailored therapeutic strategies that consider the specific immune challenges presented by coinfections. Moreover, the cytokine modulation observed offers insights into potential immunotherapeutic targets, particularly in enhancing disease management strategies for coinfected individuals. To extend this research, future studies should focus on larger, more diverse cohorts to validate our findings and explore the mechanistic bases of these immune interactions. Longitudinal studies could also provide deeper insights into how these cytokine profiles evolve with disease progression and treatment, potentially guiding more effective clinical interventions."

**Comment 6:** The authors should include the challenges associated with diagnosis, treatment, and control of the disease and how their finding can help in achieving this objective

Hossain MS, Shabir S, Toye P, Thomas LF, Falcone FH. Insights into the diagnosis,

vaccines, and control of Taenia solium, a zoonotic, neglected parasite. Parasit Vectors. 2023 Oct 24;16(1):380. doi: 10.1186/s13071-023-05989-6.

**Response:** Thank you. We have added the challenges associated with diagnosis, treatment, and control of NCC and how our findings can help in achieving this objective.

Placed in Introduction section: Line 140 - 146

Diagnosing NCC accurately is a challenge due to its varied presentations and the limitations of diagnostic tools such as ELISA, with sensitivity varying across different disease stages and cyst characteristics (6–8). Despite the high specificity of the anitbody-ELISA test for T. solium, confirming exposure, its inability to differentiate between active and inactive infections highlights the need for more definitive diagnostic approa692ches (9). While most Ag-ELISA tests are effective in detecting active infection and for monitoring cyst clearance post treatment, they are unable to detect inactive cysts, calcified cysts and single cysts (4,9).

**Minor Comments:**

1. **Comment:** Follow the scientific nomenclature to write the scientific names- See the line number 34 in the abstract.

**Response:** Thank you. We have corrected the name to follow scientific nomenclature” *Taenia solium*”.

2. **Comment:** Check the citation style**-** one style should be used throughout the manuscript

**Response:** Thank you. We adopted a single style of citing which is Vancouver style.

3. **Comment:** Re-check the data for any typological error.

**Response:** Thank you. We have rechecked and corrected all identified typological errors.

**Reviewer #2 Comments and Responses:**

**Comment 1:** In the methods section, the description of the study groups is not clear; the text, in general, needs to be improved. In the methodology section, it is not explained why they decided to evaluate the presence of eggs or proglottids in faeces (taeniasis).

**Response:** We have rewritten the methodology section giving more details into how the groups were formed, how each test was selected and how the participants were included.

**Methodology section,** Page. 5 (lines 195 - 214).

Although the large study also collected stool samples for evaluation of eggs and proglottids, the scope of this study did not include this evaluation. To avoid confusion, we have removed the information regarding stool-based egg and proglottid evaluation from the methods section.

1. **Comment:** In Authors summary Line 61-62: “Neurocysticercosis is caused by the larval stage of tapeworm Taenia solium and is associated with seizures and other.”

**Response:** We have rewritten the author summary following changes in the discussion and results section.

2. **Comment:** Line 64: Indicate which regions.

**Response:** We have added “sub-Saharan Africa".

3. **Comment:** Line 78: “The ingestion of eggs shed in the stool of a human tapeworm carrier leads to the lodging of Taenia solium larvae in the central nervous system, causing NCC”. This expression is not entirely correct, parasites can establish and develop in muscles, connective tissue, eyes, and brain.

**Response:** Thank you for pointing this out. We have now amended this sentence accordingly. See Introduction section, Paragraph 1, Line 129.

4. **Comment: Line: 80: Change invade to penetrate**

**Response:** Thank you for this point. However, we have updated the introduction section and this was also resolved as part of the new introduction section.

See Introduction section: Paragraph 1.

5. **Comment: Line 94: Which cells?**

Response: Immune Cells (CD4+ T cells, CD8+ T cells, B cells, macrophages, and dendritic cells) Please see Introduction section, page. 4. Paragraph 1, Line 164.

6. **Comment:** Line 129: Missing Reference?

**Response:** We have added the respective citation (https://doi.org/10.2307/2986303).

**General Comments:**

**Comment 1:** It is necessary for the authors to rewrite the text before publication, it needs considerable improvement.

**Response:** Thank you for your observation, we have now rewritten majority of the text as shown in track changes in the revised manuscript. We have also rewritten the results and discussion section.

**Comment 2:** The discussion section is far too long, in part because the authors comment on numerous small differences among groups rather than focusing on those that are well-supported by the data, i.e., those that are statistically significant.

**Response:** We agree with the reviewer. This is the first study of its kind aiming to integrate clinical, radiological, and immunological findings in a unique and carefully selected population of HIV+ and HIV- people living in rural SSA. Thus, we also felt it necessary to discuss the other aspects besides the immunological findings in greater detail. Indeed, this was also asked to be done by the second reviewer. Nevertheless, we have improved and cut-down the discussion.

**Comment 3:** The major issue with this manuscript is that the conclusions are overstated. Differences among groups that are not statistically significant should not be presented as conclusions. The actual p-values should be included in figures where the authors claim there is a difference, and the discussion should help readers understand the limitations of the sample sizes and effect sizes that contribute to the uncertainty in the findings.

**Response:**

Thank you for your critical feedback on the conclusions drawn in our manuscript. We acknowledge the importance of presenting statistically robust findings and appreciate your guidance on enhancing the clarity and accuracy of our conclusions.

**Amendments Made:**

**Statistical Analysis Revisions:** Following your comments, we have reviewed our data and applied more stringent statistical methods. Specifically, we have revised our approach to include adjustment for multiple comparisons where applicable, and we have included exact p-values in our figures and results section to transparently report the statistical significance of our findings. These changes are reflected on pages 8 to 16 of the manuscript.

**Revised Conclusion:** We have carefully rewritten the conclusions to more accurately reflect the data without overstating the implications. The new conclusion text, located in the Conclusion section on page 20, reads:

"Our findings suggest that coinfection with HIV and neurocysticercosis (NCC) does not significantly modulate peripheral cytokine profiles, despite the elevated IL-5 levels observed in HIV-positive individuals with cysticercosis. While this observation hints at potential immunological interplay, the lack of statistical significance in broader cytokine modulation warrants cautious interpretation. Notably, the higher cysticercosis antigen positivity among HIV-positive participants raises biological implications for NCC progression in the context of HIV coinfection. However, our results underscore the need for robust, statistically significant data to draw conclusive statements about the immunological impacts of coinfection. Future studies with larger sample sizes and advanced statistical analyses are warranted to further elucidate these interactions and inform the understanding and management of coinfected patients."

**Comment 4:** The results section needs improvement in terms of presentation of results. The narrative is contradictory in several sections.

**Response:** Thank you for your constructive feedback regarding the presentation of results in our manuscript. We recognize the importance of clear and consistent reporting, and we appreciate your pointing out the areas where our narrative might have seemed contradictory.

We have thoroughly reviewed and revised the entire Results section to ensure clarity and consistency in the presentation of our findings.

**Tables to support Figures 1 – 4 included in the Manuscript.**

| **Table 1** |  |  |  |  |  |  |  |  |  |  |  |  |
| --- | --- | --- | --- | --- | --- | --- | --- | --- | --- | --- | --- | --- |
| **Cytokine** | **HIV+CC+** | | **HIV+CC-** | | **p-values** | **adj p-values** | **HIV-CC+** | | **HIV-CC-** | | **p-values** | **adj p-values** |
|  | **Conc** | **IQR** | **Conc** | **IQR** |  |  | **Conc** | **IQR** | **Conc** | **IQR** |  |  |
| TNF-a | 3.905 | 1.71 - 7.325 | 3.184 | 1.215 - 6.69 | 0.3155 | 1 | 4.407 | 1.592 - 7.06 | 6.737 | 2.416 - 11.54 | 0.0485 | 1 |
| IL-06 | 2.933 | 1.728 - 4.307 | 2.838 | 1.728 - 4.835 | 0.7578 | 1 | 2.886 | 1.807 - 4.227 | 4.102 | 1.592 - 9.413 | 0.2321 | 1 |
| IL-8 | 60.12 | 11.77 - 309.1 | 49.45 | 15.0 - 209.5 | 0.6998 | 1 | 49.21 | 19.67 - 578.6 | 103.6 | 21.46 - 1621.0 | 0.1564 | 1 |
| IL-10 | 1.661 | 0.8673 - 2.706 | 2.251 | 1.181 - 4.239 | 0.0574 | 1 | 1.531 | 1.181 - 2.608 | 2.419 | 0.9954 - 4.64 | 0.0986 | 1 |
| IL-1b | 6.555 | 4.557 - 16.42 | 8.472 | 4.1 - 16.42 | 0.3977 | 1 | 6.896 | 4.232 - 15.77 | 12.08 | 5.256 - 17.58 | 0.0351 | 1 |
| IFN-g | 33.29 | 20.14 - 48.6 | 22.87 | 10.56 - 48.6 | 0.1634 | 1 | 29.71 | 16.45 - 48.6 | 38.26 | 24.2 - 48.6 | 0.0366 | 1 |
| IL-4 | 26.29 | 13.72 - 50.83 | 21.58 | 16.26 - 49.2 | 0.4274 | 1 | 43.51 | 16.26 - 77.63 | 52.82 | 17.51 - 107.0 | 0.2142 | 1 |
| IL-17 | 6.271 | 4.743 - 8.765 | 6.271 | 4.743 - 9.892 | 0.7274 | 1 | 8.073 | 5.156 - 10.76 | 7.996 | 4.846 - 14.21 | 0.5292 | 1 |
| IL-13 | 297.7 | 239.9 - 458.4 | 329.8 | 239.9 - 472.5 | 0.1992 | 1 | 344.1 | 239.9 - 457.9 | 397.1 | 223.6 - 472.5 | 0.7585 | 1 |
| IL-5 | 6.132 | 4.793 - 6.132 | 4.245 | 2.431 - 7.325 | 0.6511 | 1 | 3.673 | 2.431 - 6.132 | 6.132 | 6.132 - 7.325 | 0.0008 | 0.0336 |
| VCAM-1 | 817022 | 396012.0 - 1286345.0 | 674700 | 320800.0 - 907410.0 | 0.0735 | 1 | 587446 | 218775.0 - 779692.0 | 595909 | 443317.0 - 894483.0 | 0.1463 | 1 |
| ICAM-1 | 246996 | 118900.0 - 491587.0 | 169500 | 94730.0 - 343322.0 | 0.087 | 1 | 252584 | 127517.0 - 556238.0 | 257361 | 77065.0 - 474422.0 | 0.4847 | 1 |
| IL-12 | 325.1 | 159.7 - 526.5 | 248.2 | 65.62 - 503.5 | 0.1061 | 1 | 378.2 | 137.2 - 531.7 | 449.5 | 173.8 - 611.6 | 0.2374 | 1 |
| IL-18 | 278.1 | 182.8 - 444.3 | 259.4 | 185.1 - 395.2 | 0.6998 | 1 | 245.5 | 128.8 - 328.3 | 229.4 | 154.9 - 298.9 | 0.1564 | 1 |
|  |  |  |  |  |  |  |  |  |  |  |  |  |

| **Table 2** |  |  |  |  |  |  |  |  |  |  |  |  |
| --- | --- | --- | --- | --- | --- | --- | --- | --- | --- | --- | --- | --- |
| **Cytokine** | **HIV+NCC+** | | **HIV+ NCC-** | | **p-value** | **adj p-values** | **HIV- NCC+** | | **HIV- NCC-** | | **p-value** | **adj p-values** |
|  | **Conc** | **IQR** | **Conc** | **IQR** |  |  | **Conc** | **IQR** | **Conc** | **IQR** |  |  |
| TNF-a | 3.905 | 1.215 - 6.831 | 3.703 | 1.71 - 7.426 | 0.7684 | 1 | 4.978 | 1.592 - 8.939 | 7.056 | 2.993 - 11.26 | 0.1497 | 1 |
| IL-06 | 2.279 | 1.455 - 5.124 | 2.49 | 1.568 - 4.307 | 0.6462 | 1 | 3.12 | 2.002 - 4.13 | 3.881 | 2.039 - 8.411 | 0.4397 | 1 |
| IL-8 | 38.91 | 11.98 - 75.74 | 52.95 | 11.87 - 260.6 | 0.7565 | 1 | 60.95 | 29.11 - 1111.0 | 176.9 | 28.19 - 1403.0 | 0.4932 | 1 |
| IL-10 | 1.97 | 0.9735 - 2.586 | 1.764 | 1.077 - 4.215 | 0.9345 | 1 | 1.408 | 1.061 - 4.239 | 2.419 | 1.2 - 4.586 | 0.1304 | 1 |
| IL-1b | 6.055 | 4.1 - 15.11 | 7.056 | 4.557 - 16.42 | 0.5268 | 1 | 7.557 | 4.628 - 16.42 | 9.474 | 5.587 - 16.95 | 0.555 | 1 |
| IFN-g | 33.29 | 18.95 - 48.6 | 30.91 | 14.74 - 48.6 | 0.9085 | 1 | 22.87 | 16.11 - 48.6 | 39.2 | 21.46 - 49.64 | 0.21 | 1 |
| IL-4 | 26.29 | 15.51 - 43.29 | 26.29 | 15.88 - 50.92 | 0.6111 | 1 | 39.32 | 15.78 - 90.05 | 56.3 | 21.37 - 112.6 | 0.1866 | 1 |
| IL-17 | 6.271 | 4.743 - 8.765 | 6.271 | 4.743 - 9.892 | 0.8877 | 1 | 8.709 | 6.271 - 9.892 | 8.073 | 5.156 - 13.29 | 0.6687 | 1 |
| IL-13 | 261.7 | 223.6 - 458.4 | 300.5 | 239.9 - 472.5 | 0.2471 | 1 | 358.4 | 270.6 - 472.5 | 391.8 | 239.9 - 472.5 | 0.8309 | 1 |
| IL-5 | 6.132 | 2.431 - 6.132 | 6.132 | 3.071 - 7.325 | 0.207 | 1 | 3.661 | 3.071 - 7.325 | 6.132 | 3.816 - 7.325 | 0.1458 | 1 |
| VCAM-1 | 1052381 | 589064.0 - 1472149.0 | 691719 | 425214.0 - 986247.0 | 0.1823 | 1 | 599400 | 315200.0 - 769193.0 | 590087 | 424023.0 - 876335.0 | 0.6399 | 1 |
| ICAM-1 | 227300 | 65866.0 - 445608.0 | 208300 | 104865.0 - 447643.0 | >0.9999 | 1 | 258700 | 136211.0 - 530854.0 | 272403 | 90733.0 - 570044.0 | 0.8677 | 1 |
| IL-12 | 271.2 | 129.7 - 503.5 | 360.3 | 100.3 - 503.5 | 0.7628 | 1 | 404.4 | 190.1 - 503.5 | 503.5 | 253.9 - 658.7 | 0.1804 | 1 |
| IL-18 | 263.5 | 215.0 - 387.3 | 294.9 | 194.0 - 454.6 | 0.8217 | 1 | 205.2 | 126.8 - 299.9 | 250.5 | 157.8 - 346.7 | 0.1831 | 1 |
|  |  |  |  |  |  |  |  |  |  |  |  |  |

| **Table 3** |  |  |  |  |  |  |  |  |  |  |  |  |
| --- | --- | --- | --- | --- | --- | --- | --- | --- | --- | --- | --- | --- |
| **Cytokine** | **Active** | | **Inactive** | | **p-value** | **adj p-values** |  |  |  |  |  |  |
|  | **Conc** | **IQR** | **Conc** | **IQR** |  |  |  |  |  |  |  |  |
| TNF-a | 5.95 | 2.545 - 9.505 | 4.095 | 1.234 - 6.445 | 0.1757 | 1 |  |  |  |  |  |  |
| IL-06 | 3.12 | 2.03 - 11.85 | 2.279 | 1.543 - 5.83 | 0.4981 | 1 |  |  |  |  |  |  |
| IL-8 | 55.25 | 13.43 - 600.9 | 46.64 | 16.71 - 418.6 | 0.8596 | 1 |  |  |  |  |  |  |
| IL-10 | 1.918 | 1.131 - 2.074 | 1.603 | 1.017 - 4.053 | 0.9751 | 1 |  |  |  |  |  |  |
| IL-1b | 6.396 | 5.402 - 9.821 | 6.555 | 4.1 - 16.42 | 0.731 | 1 |  |  |  |  |  |  |
| IFN-g | 28.08 | 17.14 - 48.2 | 33.29 | 18.21 - 48.6 | 0.8924 | 1 |  |  |  |  |  |  |
| IL-4 | 41.34 | 22.06 - 63.24 | 26.29 | 15.65 - 41.57 | 0.3526 | 1 |  |  |  |  |  |  |
| IL-17 | 8.419 | 6.781 - 10.07 | 6.271 | 4.743 - 9.222 | 0.1362 | 1 |  |  |  |  |  |  |
| IL-13 | 299.1 | 256.2 - 368.1 | 300.5 | 239.9 - 472.5 | 0.8428 | 1 |  |  |  |  |  |  |
| IL-5 | 3.071 | 1.634 - 6.132 | 6.132 | 3.071 - 7.325 | 0.0389 | 0.5057 |  |  |  |  |  |  |
| VCAM-1 | 630583 | 387200.0 - 1607345.0 | 738308 | 560449.0 - 1238318.0 | 0.8518 | 1 |  |  |  |  |  |  |
| ICAM-1 | 538615 | 292237.0 - 823903.0 | 197836 | 46463.0 - 299200.0 | 0.0059 | 0.0826 |  |  |  |  |  |  |
| IL-12 | 315 | 213.1 - 492.0 | 404.4 | 144.7 - 503.5 | 0.8922 | 1 |  |  |  |  |  |  |
| IL-18 | 255.7 | 217.8 - 327.3 | 243.3 | 148.9 - 423.2 | 0.8845 | 1 |  |  |  |  |  |  |
|  |  |  |  |  |  |  |  |  |  |  |  |  |

| **Table 4** |  |  |  |  |  |  |  |  |  |  |  |  |
| --- | --- | --- | --- | --- | --- | --- | --- | --- | --- | --- | --- | --- |
| **Cytokine** | **HIV+Active** | | **HIV+Inactive** | | **p-value** | **adj p-values** | **HIV-Active** | | **HIV-Inactive** | | **p-value** | **adj p-values** |
|  | **Conc** | **IQR** | **Conc** | **IQR** |  |  | **Conc** | **IQR** | **Conc** | **IQR** |  |  |
| TNF-a | 8.939 | 5.368 - 11.92 | 2.641 | 1.215 - 4.273 | 0.0034 | 0.0952 | 2.545 | 2.069 - 7.004 | 6.445 | 2.962 - 12.1 | 0.3841 | 1 |
| IL-06 | 7.141 | 2.189 - 29.32 | 2.002 | 1.527 - 3.364 | 0.1104 | 1 | 3.12 | 2.021 - 3.12 | 4.13 | 1.543 - 16.38 | 0.3447 | 1 |
| IL-8 | 49.56 | 12.17 - 2062.0 | 34.33 | 11.82 - 74.28 | 0.4562 | 1 | 60.95 | 25.36 - 121.9 | 51.78 | 20.24 - 2870.0 | 0.6064 | 1 |
| IL-10 | 1.97 | 1.225 - 7.247 | 1.632 | 0.8967 - 3.548 | 0.7033 | 1 | 1.817 | 0.977 - 2.026 | 1.408 | 1.121 - 4.403 | 0.6788 | 1 |
| IL-1b | 8.058 | 5.306 - 21.7 | 5.42 | 4.059 - 12.59 | 0.1686 | 1 | 5.683 | 4.627 - 7.147 | 16.42 | 10.99 - 62.31 | 0.0539 | 1 |
| IFN-g | 47.6 | 27.32 - 54.74 | 27.32 | 10.15 - 37.26 | 0.1073 | 1 | 17.48 | 11.91 - 36.43 | 48.6 | 27.62 - 51.67 | 0.1454 | 1 |
| IL-4 | 43.29 | 13.25 - 79.89 | 21.27 | 10.91 - 38.29 | 0.2225 | 1 | 39.4 | 22.31 - 64.85 | 39.32 | 16.02 - 109.3 | 0.6738 | 1 |
| IL-17 | 6.95 | 6.5 - 12.45 | 6.271 | 3.726 - 6.95 | 0.0949 | 1 | 8.765 | 7.172 - 9.892 | 8.765 | 4.743 - 14.82 | 0.978 | 1 |
| IL-13 | 297.7 | 222.2 - 1197.0 | 250.8 | 212.1 - 322.5 | 0.49 | 1 | 300.5 | 270.2 - 377.8 | 472.5 | 331.4 - 472.5 | 0.0679 | 1 |
| IL-5 | 6.132 | 1.634 - 6.132 | 6.132 | 2.591 - 6.132 | 0.5784 | 1 | 3.071 | 2.352 - 3.071 | 6.132 | 3.661 - 7.325 | 0.0225 | 0.585 |
| VCAM-1 | 1472149 | 479648.0 - 3019623.0 | 895344 | 315675.0 - 1366250.0 | 0.3284 | 1 | 529500 | 328500.0 - 630583.0 | 649600 | 560449.0 - 810929.0 | 0.1898 | 1 |
| ICAM-1 | 445608 | 73028.0 - 680084.0 | 180684 | 36761.0 - 302378.0 | 0.3399 | 1 | 719400 | 432211.0 - 1016145.0 | 197836 | 72304.0 - 324300.0 | 0.007 | 0.189 |
| IL-12 | 271.2 | 155.0 - 746.2 | 174.9 | 81.64 - 503.5 | 0.4869 | 1 | 332.7 | 171.8 - 553.6 | 503.5 | 364.8 - 536.3 | 0.3621 | 1 |
| IL-18 | 263.5 | 203.0 - 1326.0 | 246.7 | 196.9 - 393.5 | 0.799 | 1 | 249.6 | 165.1 - 299.1 | 205.2 | 121.2 - 456.8 | >0.9999 | 1 |
