# Supplementary material for: Evaluating the modulation of peripheral immune profile in people living with HIV and (Neuro)cysticercosis
Source: PLoS Negl Trop Dis. 2024 Aug 2;18(8):e0012345. doi: 10.1371/journal.pntd.0012345 (PMC11324146; doi:10.1371/journal.pntd.0012345)
Supplement: Attachment — Submitted filename: Response to Reviewers.docx [file pntd.0012345.s007.docx]

**Point-by-point responses to reviewers’ comments**

**Manuscript title:** Evaluating the modulation of peripheral immune profile in People Living with HIV and Neurocysticercosis.

**Manuscript Number:** PNTD-D-23-01498

**Reviewer Comments and Responses**

**Minor Comments:**

**Comment 1: Tables 1 and 3:**

- Transfer these tables to the supplementary material.

**Response:** We appreciate the reviewer's feedback regarding Tables 1 and 3. In response, we have transferred these tables to the supplementary material and summarized the key information in the main text as follows:

For Table 1,now S1_demographics, we have integrated the baseline demographic and clinical characteristics of the study participants into the main text. Specifically, the main findings are that the study included 234 participants, with a median age of 43 years, and a male predominance (65%). HIV+ participants had a higher proportion of positive cysticercosis antigen results (43.6%) compared to HIV- participants (28.2%).

For Table 3, now S2_regression table, the detailed cytokine profile data and the results of the multiple regression analysis have been summarized. Notably, we found significant associations between HIV status and increased levels of TNF-α, IL-8, IFN-γ, IL-17, IL-12, IL-6, and IL-5, while cysticercosis status was associated with increased IL-5 and decreased IL-10 levels.

We believe these changes improve the readability of the manuscript while preserving the essential information.

**Comment 2: Figures**

- Figures would be better expressed in the text with standard deviations.

**Response:** Thank you very much for this insightful comment. In this study, we evaluated the differences between groups using the Mann-Whitney U test due to the non-parametric nature of the data. Consequently, we have now reported the Standard Error of the Mean (SEM) instead of the standard deviation to better represent the variability and reliability of the data. The SEM provides a more accurate measure of variability for non-parametric data and aligns with the statistical methods employed. We have updated the figure descriptions and the results section to reflect this change, and the detailed data supporting these figures are now provided in the supplementary material.

**Comment 3: Statistical Significance:**

- Reviewers note that unless the p-value for trends lies between 0.05 and 0.1, it is difficult to support the concept that the trend could become clinically significant.

**Response:** We appreciate this important comment regarding the interpretation of p-values for trends. In the revised manuscript, we have carefully reviewed the p-values for all cytokine comparisons. For the cytokine ICAM-1, the adjusted p-value is 0.0826, which falls within the range of 0.05 to 0.1, indicating a tendency that could potentially become clinically significant. We have explicitly mentioned this trend in the results section and the figure legend for Figure 3 to highlight the observed tendency towards increased ICAM-1 levels in patients with radiologically active NCC. This adjustment ensures that our findings are presented with the appropriate statistical context and provides a clearer interpretation of potential clinical significance.

Following this comment we have also corrected the discussion part which addresses the trend to show the only trend observed was on ICAM-1.
